# Pandemic influenza in Australia: Using telephone surveys to measure perceptions of threat and willingness to comply

**DOI:** 10.1186/1471-2334-8-117

**Published:** 2008-09-15

**Authors:** Margo Barr, Beverley Raphael, Melanie Taylor, Garry Stevens, Louisa Jorm, Michael Giffin, Sanja Lujic

**Affiliations:** 1 Centre for Epidemiology and Research, New South Wales Department of Health, Sydney, Australia; 2 School of Medicine, University of Western Sydney, Sydney, Australia

## Abstract

**Background:**

Baseline data is necessary for monitoring how a population perceives the threat of pandemic influenza, and perceives how it would behave in the event of pandemic influenza. Our aim was to develop a module of questions for use in telephone health surveys on perceptions of threat of pandemic influenza, and on preparedness to comply with specific public health behaviours in the event of pandemic influenza.

**Methods:**

A module of questions was developed and field tested on 192 adults using the New South Wales Department of Health's in-house Computer Assisted Telephone Interviewing (CATI) facility. The questions were then modified and re field tested on 202 adults. The module was then incorporated into the New South Wales Population Health Survey in the first quarter of 2007. A representative sample of 2,081 adults completed the module. Their responses were weighted against the state population.

**Results:**

The reliability of the questions was acceptable with kappa ranging between 0.25 and 0.51. Overall 14.9% of the state population thought pandemic influenza was very or extremely likely to occur; 45.5% were very or extremely concerned that they or their family would be affected by pandemic influenza if it occurred; and 23.8% had made some level of change to the way they live their life because of the possibility of pandemic influenza. In the event of pandemic influenza, the majority of the population were willing to: be vaccinated (75.4%), be isolated (70.2%), and wear a face mask (59.9%). People with higher levels of threat perception are significantly more likely to be willing to comply with specific public health behaviours.

**Conclusion:**

While only 14.9% of the state population thought pandemic influenza was very or extremely likely to occur, a significantly higher proportion were concerned for self and family should a pandemic actually occur. The baseline data collected in this survey will be useful for monitoring changes over time in the population's perceptions of threat, and preparedness to comply with specific public health behaviours.

## Background

If an outbreak of pandemic influenza should occur, it is essential that public health authorities are prepared to act. While resources have been prepared to educate the population about the nature of a threat and planned government actions,[1] it is necessary to understand the potential response of a population.

Most of the existing information about a population's response to the threat of pandemics comes from research on outbreaks of the SARS coronavirus, most notably in Hong Kong, Singapore, and Canada, [2-5] and on studies of risk perception and anticipated behaviours in a potential pandemic in humans from the avian influenza virus (especially the H5N1 subtype). [6-9] To date, Australia has been relatively unaffected by SARS or H5N1; however, some of Australia's neighbours have experienced limited outbreaks: for example, SARS in Hong Kong and Singapore; and H5N1 in Indonesia and Hong Kong and China. Globally, the threat of a pandemic of H5N1 is high.

A key component of a population's response is the perception of risk or threat. Research shows that in a SARS outbreak willingness to comply with risk-reducing behaviours is linked to the perceived immediacy and seriousness of the threat.[2,3,5] Three risk perception studies on potential avian influenza outbreaks were conducted in 2005. In the first study, Lau et al. surveyed residents of Hong Kong on a potential outbreak of H5N1.[7] Their study focussed on protective behaviours and likely compliance with them; however, the researchers also asked respondents about the perceived threat of H5N1 and the likelihood of it occurring within the next 12 months. It was found that 33% of respondents felt the chance of an outbreak was high or very high. Lau's study also asked respondents how worried they would be about oneself or a family member contracting the virus in the event of a local outbreak; 54% said they would be very worried.

In the second study, de Zwart et al. compared the risk perceptions of European and Asian respondents to the threat of avian influenza,[8] and measured self-efficacy beliefs to assess the likely compliance with protective health measures. Overall the study found that 45% of respondents thought they were likely or very likely to become infected should an outbreak of avian influenza occur. This figure varied from 32% (Denmark and Singapore) to 61% (Poland and Spain). The researchers took a composite measure of risk perception and found that higher scores were observed in Europe rather than Asia. They found higher risk perceptions in females and older respondents; while lower self-efficacy beliefs in Europe suggested that adherence to protective measures would be lower in Europe.

In the third study, Di Giuseppe et al. surveyed the knowledge and attitudes of an Italian population to avian influenza.[9] They found that around 19% of respondents had a high risk perception and felt very much at risk of contracting avian influenza. In this study lower socioeconomic status and lower education levels were associated with higher risk perception, and those with a higher risk perception were more likely to comply with hygiene practices to avoid the spread of disease.

Our aim was to develop a module of questions for use in telephone health surveys on perceptions of threat of pandemic influenza, and on preparedness to comply with specific public health behaviours in the event of pandemic influenza.

## Methods

### Question design

A literature search was conducted to identify existing tools for collecting information on perceptions of pandemic influenza with the underlying themes of likelihood, effect on family, life changes, and compliance with government authorities. The abovementioned studies by Lau et al. and de Zwart et al. and Di Giuseppe et al. had not been reported when our literature search was conducted. [7-9] As such, our literature search identified no relevant studies on response to pandemic influenza specifically, although other studies have been published on general threat perception and compliance with protective behaviours in the context of infectious diseases or other emergencies.

The primary reference was a study by Canadian researchers on anticipated public response to terrorism.[10] Questions on the threat likelihood, effect on family, and behavioural compliance, were adapted with permission by subject matter experts and survey methodologists. Each proposed question was considered for clarity, ease of administration, and possible biases. A set of 6 questions was developed for field-testing (Table 1), as well as an additional open question: "Do you have any comments you would like to make on any of the questions or any other issues?"

**Table 1:** Field testing for reliability and convergent validity, including original wording of questions at first test, changes made, and revised wording at second test

**First Field Test Initial question and response summary**	**Changes made**	**Second Field Test Final question and response summary**
Q.1 How likely do you think it is that pandemic influenza will occur in Australia? (*Not likely, somewhat likely, very likely, extremely likely, don't know*) Weighted kappa = 0.43 (0.33–0.54) Agreement = 61% Don't know = 2.1% Refused = 0%	None to question. Response altered to a Likert scale: increased from 4 to 5 options.	Q.1 How likely do you think it is that pandemic influenza will occur in Australia? (*Not at all likely, a little likely, moderately likely, very likely, extremely likely, don't know*) Weighted kappa = 0.38 (0.28–0.47) Indicator kappa = 0.44 (0.30–0.58) Agreement = 45.5% Don't know = 3.9% Refused = 0%

Q.2 How likely do you think it is that you or your family would be directly affected by an influenza pandemic in Australia? (*Not likely, somewhat likely, very likely, extremely likely, don't know)* Weighted kappa = 0.48 (0.38–0.58) Agreement = 62% Don't know = 2.6% Refused = 0%	Additional context added before question to provide better context. Likelihood of being affected was changed to concern about being affected, to tap a sense of vulnerability rather than probability. Responses altered to reflect concern and increase to 5 options.	Q.2 If an influenza pandemic were to occur in Australia, how concerned would you be that you or your family would be directly affected by it? *(Not at all concerned, a little concerned, moderately concerned, very concerned, extremely concerned, don't know)* Weighted kappa = 0.28 (0.18–0.38) Indicator kappa = 0.25 (0.12–0.38) Agreement = 39.1% Don't know = 0% Refused = 0%

Q.3 Do you feel you have changed the way you live your life because of the possibility of an influenza pandemic in Australia? *(Yes/No)* Kappa = 0.51 (0.34–0.68) Agreement = 87% Don't know = 0% Refused = 0%	Changed from a binary Yes/No question to a Likert scale response assessing the degree to which respondents had made any change. Question wording was altered to reflect that change.	Q.3 How much have you changed the way you live your life because of the possibility of an influenza pandemic? *(Not at all, a little, moderately, very much, extremely, don't know)* Weighted kappa = 0.34 (0.24–0.44) Indicator kappa = 0.43 (0.30–0.56) Agreement = 57.5% Don't know = 1.5% Refused = 0%

Q.4 In case of an emergency situation, government authorities might request cooperation from the public in a number of ways. Please indicate how willing would you be to do the following: Receive vaccination? (*not willing, somewhat willing, very willing, extremely willing don't know, refused*) Weighted kappa = 0.39 (0.28–0.50) Agreement = 53% Don't know = 2.1% Refused = 0%	Additional context added to the introduction to specify pandemic influenza and make questions more relevant. Response altered to a Likert scale: increased from 4 to 5 options.	Q.4 In case of an emergency situation such as an influenza pandemic, government authorities might request cooperation from the public in a number of ways Please indicate how willing would you be to receive vaccination? (*not at all willing, a little willing, moderately willing, very willing, extremely willing don't know, refused*) Weighted kappa = 0.45 (0.35–0.56) Indicator kappa = 0.51 (0.34–0.67) Agreement = 57.1% Don't know = 0% Refused = 0%

Q.5 Isolate yourself from others? (*not willing, somewhat willing, very willing, extremely willing don't know, refused*) Weighted kappa = 0.51 (0.42–0.60) Agreement = 56% Don't know = 1.0% Refused = 0%	Question stem repeated. Response altered to a Likert scale: increased from 4 to 5 options.	Q.5 How willing would you be to isolate yourself from others if needed? (*not at all willing, a little willing, moderately willing, very willing, extremely willing don't know, refused*) Weighted kappa = 0.4 (0.31–0.5) Indicator kappa = 0.48 (0.33–0.62) Agreement = 52.7% Don't know = 1.0% Refused = 0%

Q.6 ...Wear a face mask? (*not willing, somewhat willing, very willing, extremely willing don't know, refused*) Weighted kappa = 0.50 (0.40–0.60) Agreement = 56% Don't know = 1.0% Refused = 0%	Question stem repeated. Response altered to a Likert scale: increased from 4 to 5 options.	Q.6 How willing would you be to wear a face mask? (*not at all willing, a little willing, moderately willing, very willing, extremely willing don't know, refused*) Weighted kappa = 0.48 (0.39–0.56) Indicator kappa = 0.51 (0.39–0.63) Agreement = 45.8% Don't know = 0.5% Refused = 0%

Source: New South Wales Health Survey Program. Sydney: New South Wales Department of Health, 2008.

### Field testing

The pandemic influenza questions were field tested for test-retest reliability using the protocol of the New South Wales Health Survey Program.[11] The questions were then modified based on the results from the field testing and were re field tested. For both field tests the target sample was 200 persons living in the state aged 16 years and over stratified by geographical region. This sample size ensures that a kappa of 0.6 (good or excellent) is able to be detected at a significance level of 5% and a power of 80% when compared to a kappa of 0.4 or less (fair or poor) for response frequencies greater than 20%.[11]

Households were contacted using random digit dialling. One person aged 16 years and over from each household was randomly selected for field testing. Trained interviewers conducted the interviews. Up to 7 calls were made to establish initial contact with a household, and at least 5 calls were made to contact a selected respondent. When the respondent completed the first field test, an appointment was made for a retest at least a week later but within 3 weeks of the initial field test. If a respondent was unable to be contacted during this 2 week window they were deemed to be unavailable and their initial field test was deleted.[11]

Test-retest reliability and validity were estimated by Cohen's kappa statistic for binary variables, and weighted kappa with Cicchetti-Allison weights for ordinal variables. Unbalanced tables were corrected using the method described by Crewson.[11] Since erroneously low values of kappa can arise from skewed data, per cent agreement was also presented for categorical variables, calculated as the proportion of respondents in the same category at test and retest. Responses for don't know and refused are also reviewed.[11]

Data manipulation and analysis were conducted using SAS Version 9.2.[11]

### The survey

The New South Wales Population Health Survey is a continuous telephone survey of the health of the state population using the in-house CATI facility of the New South Wales Department of Health.[11] Only residential phone numbers were used in the sample, as residential phone coverage in Australia still remains high,[12] and results from persons who only have mobile phones has been shown to be comparable in the United States.[13,14]

The pandemic influenza module was administered as part of the survey between 22 January and 31 March 2007. The pandemic influenza questions were submitted to a lead ethics committee for approval prior to use. The survey also includes other modules on health behaviours, health status (including psychological distress, using the Kessler K10 measure, and self-rated health status), and access to health services, as well as the demographics of respondents and households. The target population for the survey is all state residents living in households with private telephones. Up to 7 calls were made to establish initial contact with a household, and 5 calls were made in order to contact a selected respondent.

Response categories were dichotomised into indicators of interest and don't knows and refused were removed. For the hypothetical questions – that is, likelihood of pandemic influenza, likelihood that family or self affected, willingness to comply with vaccination, isolation or wearing a face mask – the responses of extremely likely and very likely were combined into the indicator of interest. For the non-hypothetical question "changed way live because of the possibility of an influenza pandemic" responses a little, moderately, very much and extremely were combined into the indicator of interest: that is, changed life.

The survey data were weighted to adjust for probability of selection and for differing non-response rates among males and females and different age groups.[15] Data were manipulated and analysed using SAS version 9.2.[11] The SURVEYFREQ procedure in SAS was used to analyse the data and calculate point estimates and 95 per cent confidence intervals for the prevalence estimates. For pairwise comparisons of subgroup estimates, the p-value for a two-tailed test was calculated using the normal distribution probability function PROBNORM in SAS, assuming approximate normal distribution of each individual subgroup estimates with the estimated standard errors, and approximate normal distribution for the estimated difference.

## Results

### Field testing

In total, 192 residents aged 16 years and over completed the first field test and 202 residents completed the second field test. Estimates of test-retest reliability for the first and second field tests are shown in Table 1, including amendments made prior to the second test. Kappa and weighted kappa values for the questions ranged between 0.39 and 0.51 in the first field test and between 0.28 and 0.48 in the second field test. Kappa values for the indicators derived from the questions ranged between 0.25 and 0.51 in the second field test. There were low don't know response rates (0–3.9%) and no respondent refused to answer any question.

In response to the open question "Do you have any comments you would like to make on any of the questions or any other issues?": 79% made positive comments about the questions, 48.7% found the question wording easy to understand and answer, and 29.9% found the subject matter relevant and interesting. Of the respondents who had difficulty answering the questions, the main issues were: the questions were too vague (7.1%), response options were not descriptive enough (7.1%), or the topic area was difficult (6.5%).

### The survey

A total of 2,081 state residents aged 16 years and over completed the module on pandemic influenza. The overall response rate was 65%. The demographics of the weighted survey population were comparable with the Australian Bureau of Statistics 2006 Census for sex, persons born in Australia, persons who speak a language other than English, children in household, persons who live alone, and location (Table 2).[16]

**Table 2:** Comparison of the weighted pandemic influenza survey sample to the Australian population for key demographics

	**Weighted pandemic influenza survey sample %**	**2006 Australian Population^‡ ^%**	**P values^+^**
**Gender**		Based on = > 16 years	
Male	50.8	48.8	p = 0.069
Female	49.2	51.2	

**Age**		Based on = > 16 years	
16–24	15.1	15.5	p = 0.01
25–34	17.1	17.1	
35–44	19.7	18.8	
45–54	18.8	17.7	
55–64	14.3	14.0	
65–74	9.5	8.8	
75+	5.4	8.1	

**Highest formal qualification**		Based on = > 15 years	
None	6.1	7.9	p < 0.0001
School certificate	22.1	17.3	
High school certificate	17.2	22.3	
TAFE certificate/diploma	22.6	31.7	
University degree/equivalent	32.0	20.8	

Born in Australia **		Based on = > 15 years	p = 0.065
Yes	73.5	71.7	
No	26.5	28.3	

**Speak language other than English^#^**		Based on all years	
Yes	16.8	16.8	p = 0.982
No	83.2	83.2	

**Employed ^^**		Based on = > 15	
Yes	64.1	61.2	p = 0.007
No	35.9	38.8	

Children in household *** ^##^		Based on occupied dwellings	
Yes	41.5	43.3	p = 0.101
No	58.5	56.7	

**Living alone**		Based on = > 15 years	
Yes	11.1	12.5	p = 0.054
No	88.9	87.5	

**Location ^^^**		Based on all years	
Urban	70.1	68.4	p = 0.093
Rural	29.9	31.6	

Notes:^‡ ^ABS figures obtained from the ABS website at . Symbols refer to ABS data: * Excludes Australian External Territories; ** Excludes country of birth not stated; # language spoken at home. Excludes language spoken at home not stated;

## Count of persons in occupied private dwellings; *** children defined as being < 15 yrs old; ^based on 52 weeks in the year.

^^ Employed is defined as any paid work full-time or part time ^^^ urban/rural is defined by area health service for NSW population survey and is equal to the major cities category for the Australian census. ^+^chi squared

Source: New South Wales Health Survey Program. Sydney: New South Wales Department of Health, 2008.

Table 3 shows the responses to each question, including don't know and refused. The percentage of don't know or refused responses was low.

**Table 3:** Prevalence estimates for each question by response category including don't know and refused

Question	Response	%	95% LCI	95% UCI
Q.1 How likely do you think it is that pandemic influenza will occur in Australia?	Not at all	13.0	10.7	15.2
	A little	31.6	28.6	34.5
	Moderately	36.3	33.4	39.2
	Very	9.7	7.9	11.4
	Extremely	4.6	3.4	5.8
	Don't know	4.3	3.2	5.4
	Refused	0.6	0.2	1.0
Q.2 If an influenza pandemic were to occur in Australia, how concerned would you be that you or your family would be affected by it?	Not at all	5.0	3.7	6.3
	A little	21.6	19.0	24.2
	Moderately	26.5	23.8	29.2
	Very	30.4	27.7	33.2
	Extremely	13.9	11.6	16.3
	Don't know	2.0	1.2	2.8
	Refused	0.5	0.1	0.9
Q.3 How much have you changed the way you live your life because of the possibility of an influenza pandemic?	Not at all	74.6	71.8	77.5
	A little	14.0	11.6	16.4
	Moderately	5.6	4.4	6.9
	Very	2.6	1.2	3.9
	Extremely	1.0	0.5	1.5
	Don't know	1.6	0.9	2.3
	Refused	0.5	0.1	0.9
Q.4 Please indicate how willing would you be to receive vaccination?	Not at all	3.1	2.1	4.1
	A little	5.1	3.9	6.3
	Moderately	15.7	13.4	18.1
	Very	33.6	30.6	36.5
	Extremely	39.7	36.7	42.8
	Don't know	2.3	1.3	3.2
	Refused	0.5	0.1	0.9
Q.5 How willing would you be to isolate yourself from others if needed?	Not at all	4.9	3.5	6.3
	A little	5.9	4.5	7.4
	Moderately	17.8	15.5	20.2
	Very	34.4	31.4	37.4
	Extremely	33.0	30.2	35.9
	Don't know	3.4	2.2	4.6
	Refused	0.5	0.1	0.9
Q.6 How willing would you be to wear a face mask?	Not at all	7.8	6.1	9.5
	A little	9.2	7.3	11.0
	Moderately	21.6	19.0	24.2
	Very	30.5	27.6	33.3
	Extremely	27.1	24.5	29.8
	Don't know	3.3	2.2	4.4
	Refused	0.5	0.1	0.9

Source: New South Wales Health Survey Program. Sydney: New South Wales Department of Health, 2008.

Table 4 shows the indicators for pandemic influenza likely, concern for self and family, and changed life by sex, age group, demographic characteristics, and the indicators of level of psychological distress and general self-rated health status. Overall 14.9% of the population thought pandemic influenza was very or extremely likely, 45.5% were very or extremely concerned that they or their family would be affected by pandemic influenza, and 23.8% had made some (small to extreme) level of change to the way they live their life because of the possibility of pandemic influenza.

**Table 4:** Prevalence estimates and pairwise comparisons, threat perception questions, by socio-demographic subgroups and health status

**Population**	**Pandemic influenza likely** **(% very/extremely likely)**	**Concern for self/family** **(% very/extremely concerned)**	**Changed life** **(% a little/moderately/a lot/extremely)**	**Pandemic influenza likely + concerned for self/family**	**Pandemic influenza likely + concerned +life changed**
	**% (95% CI)**	**% (95% CI)**	**% (95% CI)**	**% (95% CI)**	**% (95% CI)**

**Total NSW population^‡^**	14.9 (12.8, 17.2)	45.5 (42.4, 48.6)	23.8 (20.9, 26.6)	9.9 (8.0, 11.8)	4.37 (2.8, 5.9)

**Gender**					
Male	14.2 (10.6, 17.8)	43.5 (38.6, 48.4)	23.5 (18.9, 28.0)	9.3 (6.1, 12.4)	4.8 (2.0, 7.6)
Female	15.8 (13.2, 18.3)	47.6 (43.8, 51.4)	24.0 (20.7, 27.4)	10.5 (8.5, 12.5)	4.0 (2.8, 5.2)

**Age^§^**					
16–24	**8.7 **(3.6, 13.8)*	**33.9 **(24.3, 43.5)*	28.1(18.2, 38.0)	**3.4 **(6.1, 12.4)***	**0.9 **(0, 2.2)***
25–34	9.9 (4.8, 14.9)	43.2 (34.3, 52.0)	20.5 (13.6, 27.4)	6.1 (8.5, 12.5)	2.5 (0.2, 4.8)
35–44	16.0 (9.1, 22.9)	47.9 (39.7, 56.1)	27.7 (19.8, 35.5)	10.4 (0.3, 6.5)	4.8 (0, 10.7)
45–54	17.1 (12.7, 21.5)	50.0 (43.5, 56.5)	22.0 (16.8, 27.1)	13.1 (2.4, 9.8)	7.2 (3.7, 10.6)
55–64	19.0 (14.0, 24.0)	47.4 (41.3, 53.5)	21.8 (16.5, 27.0)	13.0 (4.0, 16.8)	5.1 (1.6, 8.5)
65–74	**20.6 **(15.5, 25.7)*	46.3 (40.3, 52.4)	23.2 (17.9, 28.5)	13.7 (9.0, 17.1)	5.8 (2.4, 9.1)
75+	17.6 (11.8, 24.3)	**54.9 **(47.0, 62.8)*	19.8 (13.1, 26.6)	13.3 (8.7, 17.3)	4.9 (1.7, 8.0)

**Children in household**					
Yes	11.6 (7.3, 15.9)	46.7 (40.6, 52.8)	26.5 (20.8, 32.3)	7.8 (3.9, 11.8)	4.2 (0.6, 7.8)
No	16.6 (13.7, 19.4)	44.3 (40.2, 48.4)	22.8 (19.2, 26.3)	10.6 (8.4, 12.8)	4.5 (3.0, 6.1)

**Born in Australia**					
Yes	15.3 (12.8, 17.9)	46.4 (42.8, 50.0)	**21.8**(18.7, 25.0)*	9.9 (7.7, 12.0)	4.3 (2.4, 6.2)
No	13.9 (9.5, 18.2)	42.9 (36.5, 49.2)	**29.3 **(23.2, 35.5)	10.0 (6.2, 13.7)	4.6 (2.0, 7.2)

**Speak language other than English at home**					
Yes	13.5 (7.2, 19.8)	46.5 (37.5, 55.5)	**36.3 **(27.4, 45.3)**	10.3 (4.8, 15.9)	4.7 (1.0, 8.4)
No	15.3 (12.9, 17.6)	45.3 (42.0, 48.6)	**21.4 **(18.6, 24.3)	9.8 (7.8, 11.8)	4.3 (2.6, 6.0)

**Location**					
Urban	**13.1 **(10.1, 16.0)*	45.0 (40.8, 49.2)	24.7 (20.9, 28.5)	**8.5 **(6.0, 11.0)*	4.3 (2.1, 6.5
Rural	**19.1 **(16.3, 22.0)	46.6 (42.6, 50.5)	21.7 (18.4, 25.0)	**12.8 **(10.4, 15.3)	4.5 (3.1, 6.0)

**Living alone**					
Yes	18.7 (14.9, 22.5)	47.4 (42.6, 52.3)	**19.4 **(15.7, 23.1)*	**13.5 **(10.1, 16.9)*	4.3 (2.4, 6.0)
No	14.5 (12.1, 17.0)	45.3 (41.8, 48.8)	**24.3 **(21.2, 27.5)	**9.4 **(7.4, 11.5)	4.4 (2.7, 6.1)

**Highest formal qualification^§^**					
None	**27.4 **(18.2, 36.6)*	53.5 (44.0, 62.9)	26.1 (18.2, 34.0)	**18.3 **(11.2, 25.4)*	8.7 (3.2, 14.1)
School certificate	16.8 (12.5, 21.0)	45.9 (39.9, 51.8)	22.7 (17.8, 27.7)	9.6 (6.9, 12.3)	3.7 (2.1, 5.4)
High school certificate	11.5 (7.2, 15.9)	39.6 (31.7, 47.6)	22.1 (15.0, 29.3)	8.2 (4.6, 11.8)	3.8 (1.7, 5.9)
TAFE certificate/diploma	15.4 (10.9, 19.8)	44.1 (37.7, 50.6)	24.3 (18.4, 30.2)	9.2 (5.7, 12.6)	3.7 (1.1, 6.2)
University degree/equivalent	13.2 (8.7, 17.7)	47.6 (41.5, 53.6)	24.2 (18.5, 30.0)	10.1 (5.7, 14.4)	4.9 (0.9, 8.9)

**Employed**					
Yes	**13.5 **(10.6, 16.0)*	44.9 (40.6, 49.3)	22.5 (18.8, 26.3)	**8.5 **(6.4, 10.5)*	3.3 (1.9, 4.6)
No	**18.1 **(14.7, 21.4)	47.2 (42.7, 51.6)	24.1 (20.1, 28.2)	**12.3 **(9.6, 15.1)	5.2 (3.1, 7.3)

**Household income^§^**					
< $20 k	**21.2**(15.8, 26.6)*	45.9 (39.4, 52.3)	30.1 (23.0, 37.1)	**16.1 **(11.3, 20.8)*	7.0 (3.6, 10.4)
$20 k – $40 k	18.8 (13.8, 23.8)	47.2 (39.9, 54.6)	25.6 (18.8, 32.4)	12.5 (8.6, 16.4)	5.1 (2.3, 7.8)
$40 k – $60 k	18.9 (13.1, 24.7)	46.6 (38.7, 54.5)	24.8 (17.6, 32.0)	10.8 (6.6, 15.1)	2.3 (0.6, 3.9)
$60 k – $80 k	17.2(7.1, 27.3)	45.5 (35.0, 55.9)	19.5 (9.8, 29.2)	12.1 (2.1, 22.0)	7.4 (0, 17.0)
> $80 k	**9.0 **(5.8, 12.3)**	43.9 (37.5, 50.2)	22.0 (16.7, 27.4)	**5.2 **(2.9, 7.5)**	2.4 (1.0, 3.9)

**High psychological distress (≥ 22)^ψ^**					
Yes	21.5 (12.0, 30.9)	46.0 (35.3, 56.7)	30.0 (19.8, 40.2)	16.5 (8.0, 25.0)	8.8 (2.3, 15.4)
No	14.1 (11.4, 16.8)	45.7 (41.4, 50.0)	22.6 (18.9, 26.2)	8.5 (6.5, 10.4)	3.0 (1.9, 4.1)

**Positive self-rated health status (good, very good, excellent)**					
Yes	**13.9 **(10.5, 17.2)*	**43.7**(39.1, 48.3)*	**22.4 **(18.2, 26.5)*	**8.6 **(5.7, 11.5)*	3.7 (1.2, 6.3)
No	**23.4 **(15.3, 31.5)	**56.0 **(47.1, 64.9)	**32.7 **(23.5, 41.9)	**17.9 **(4.0, 10.1)	10.3 (3.2, 17.4)

Notes: Level of statistical significance: * p < 0.05; ** p < 0.01; *** p < 0.001. ^‡ ^Population level frequencies do not agree with Table 3 as don't know/refused responses were excluded from this analysis. ^§ ^For pairwise comparison testing in subgroups with more than two categories comparisons are made between each subgroup prevalence and the overall population prevalence. ^ψ^Psychological distress was measured using the K10. Values range from 10–50, with 'high' psychological distress considered as being ≥ 22. Source: New South Wales Health Survey Program. Sydney: New South Wales Department of Health, 2008.

**Figure 1:**
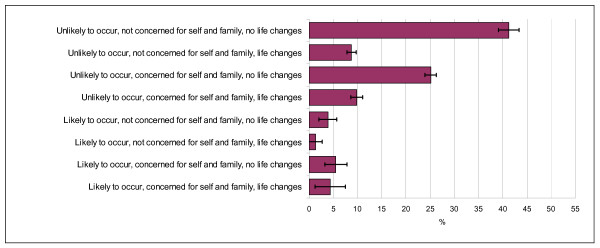
**Prevalence estimates and 95% confidence intervals for response combinations to the three questions on perceived threat for pandemic influenza**. Source: New South Wales Health Survey Program. Sydney: New South Wales Department of Health, 2008.

When the indicators for pandemic influenza likelihood, concern for self and family and changed life were combined, as shown in Figure 1, the greatest proportion of the population (41.3%) thought pandemic influenza was unlikely to occur, would not be concerned for themselves or their family, and had not changed the way they lived their life because of the possibility of pandemic influenza. A quarter of the population (25.1%) thought pandemic influenza was unlikely to occur and had not made any changes to their lives, but would be concerned for themselves and their family in the event of pandemic influenza.

Table 4 also shows the combined indicators pandemic influenza likely and concern for self and family as well as pandemic influenza likely and concern for self and family and changed life by sex, age group, demographic characteristics, and the indicators of level of psychological distress and general self-rated health status.

Table 5 shows the indicators willing to receive vaccination, isolate themselves, or wear a face mask by sex, age group, demographic characteristics, and the indicators of level of psychological distress and general self-rated health status. Overall, the majority of the population would be willing to receive vaccination (75.4%), willing to be isolated (70.2%), and willing to wear a mask (59.9%), if pandemic influenza were to occur.

**Table 5:** Prevalence estimates and pairwise comparisons, willingness to comply with health protective behaviours, by socio-demographic subgroups and health status

**Population**	**Very willing to comply with vaccination**	**Very willing to comply with isolation**	**Very willing to comply with wearing a face mask**	**Very willing to comply with ALL: vaccination + isolation+ wearing a face mask**
	**% (95%C)**	**% (95%CI)**	**% (95%CI)**	**% (95%CI)**

**Total NSW population^‡^**	75.4 (72.7, 78.1)	70.2 (67.3, 73.0)	59.9 (56.8, 63.0)	47.7 (44.5, 50.8)

**Gender**				
Male	76.2 (72.0, 80.4)	68.1 (63.5, 72.7)	59.4 (54.6, 64.3)	48.3(43.3, 53.3)
Female	74.6 (71.2, 77.9)	72.3 (68.8, 75.7)	60.4 (56.5, 64.2)	47.0 (43.2, 50.9)

**Age^§^**				
16–24	67.3 (57.8, 76.8)	**59.2 **(49.3, 69.1)*	**43.9 **(33.8, 54.1)**	**33.1 **(23.2, 43.1)**
25–34	72.3 (64.1, 80.5)	62.4 (53.7, 71.1)	53.8 (44.8, 62.8)	40.5 (31.8, 49.2)
35–44	79.3 (73.4, 85.3)	74.2 (67.4, 81.0)	66.7 (59.4, 74.0)	50.0 (41.8, 58.3)
45–54	77.8 (72.5, 83.0)	72.9 (67.1, 78.8)	61.3 (55.0, 67.6)	52.4 (45.9, 58.9)
55–64	75.8 (70.6, 80.9)	**77.4 **(72.1, 82.7)*	64.8 (58.6, 70.9)	52.4 (46.1, 58.6)
65–74	**81.5 **(77.0, 86.0)*	74.3 (69.1, 79.5)	**69.1 **(63.5, 74.7)**	**58.2 **(52.0, 64.3)**
75+	73.9 (67.4, 80.5)	72.5 (65.3, 79.6)	64.4 (56.9, 72.0)	53.4 (45.4, 61.4)

**Children in household**				
Yes	**80.5 **(75.8, 85.3)**	71.8 (66.3, 77.2)	58.4 (52.4, 64.5)	47.8 (41.6, 54.0)
No	**72.5 **(68.7, 76.3)	69.0 (65.1, 72.9)	60.4 (56.3, 64.6)	47.2 (43.0, 51.3)

**Born in Australia**				
Yes	76.5 (73.5, 79.6)	**72.0 **(68.8, 75.2)*	60.3 (56.8, 63.9)	48.6 (44.9, 52.2)
No	72.2 (66.4, 78.0)	**64.7 **(58.3, 71.2)	58.4 (51.9, 64.9)	44.9 (38.4, 51.4)

**Speak language other than English at home**				
Yes	**67.5 **(59.1, 75.9)*	**53.3 **(44.1, 62.3)***	53.4 (44.3, 62.4)	**36.9 **(28.0, 45.7)**
No	**77.0 **(74.2, 79.8)	**73.3 **(70.4, 76.2)	61.1 (57.7, 64.3)	**49.7 **(46.3, 53.1)

**Location**				
Urban	74.4 (70.7, 78.0)	68.9(64.9, 72.8)	**57.8**(53.6, 62.0)*	46.2 (41.9, 50.5)
Rural	77.6 (74.4, 80.8)	72.9 (69.4, 76.3)	**64.4 **(60.6, 68.2)	50.8 (46.7, 54.8)

**Living alone**				
Yes	72.6 (68.2, 77.0)	70.2 (65.5, 74.8)	62.0 (57.2, 66.9)	49.5 (44.5, 54.5)
No	75.8 (72.8, 78.8)	70.2 (66.9, 73.4)	59.6 (56.1, 63.1)	47.4 (43.9, 51.0)

**Highest formal qualification^§^**				
None	75.6 (67.9, 83.4)	64.0 (54.5, 73.4)	59.8 (50.6, 68.9)	42.3 (32.8, 51.8)
School certificate	71.2 (65.6, 76.8)	65.4 (59.7, 71.2)	57.2 (51.2, 63.2)	45.1 (39.1, 51.2)
High school certificate	72.9 (65.4, 80.4)	66.4 (58.6, 74.1	51.9 (43.6, 60.2)	42.1 (34.1, 50.1)
TAFE certificate/diploma	74.5 (68.7, 80.3)	73.3 (67.5, 79.1)	57.0 (50.4, 63.6)	45.7 (39.2, 52.2)
University degree/equivalent	80.4 (75.8, 85.0)	74.7 (69.4, 80.0)	**67.6 **(61.9, 73.2)*	**54.5 **(48.5, 60.5)*

**Employed**				
Yes	76.4 (72.7, 80.1)	69.5 (65.5, 73.5)	58.9 (54.5, 63.2)	47.3 (42.8, 51.7)
No	74.5 (70.6, 78.5)	71.8 (67.7, 76.0)	62.0 (57.6, 66.5)	49.1 (44.6, 53.6)

**Household income^§^**				
< $20 k	71.2 (65.2, 77.2)	71.6 (65.8, 77.4)	63.2 (56.9, 69.5)	49.9 (43.1, 56.6)
$20 k – $40 k	72.7 (66.1, 79.2)	74.3 (68.0, 80.6)	60.3 (52.8, 67.7)	47.5 (40.1, 54.8)
$40 k – $60 k	70.1 (62.5, 77.6)	67.9 (60.3, 75.5)	58.5 (50.4, 66.5)	42.1 (34.4, 49.7)
$60 k – $80 k	79.0 (70.6, 87.4)	64.3 (54.1, 74.5)	60.8 (50.7, 70.9)	46.0 (35.3, 56.6)
> $80 k	**81.4 **(76.2, 86.5)*	74.3 (68.6, 80.0)	60.5 (54.2, 66.8)	51.4 (45.0, 57.9)

**High psychological distress (≥ 22)^ψ^**				
Yes	75.6 (66.1, 85.0)	67.6 (57.3, 77.9)	55.5 (44.7, 66.4)	40.9 (30.4, 51.4)
No	77.1 (73.5, 80.7)	73.3 (69.3, 77.2)	63.1 (58.8, 67.4)	50.2 (45.8, 54.6)

**Positive self-rated health status (good, very good, excellent)**				
Yes	77.7 (73.9, 81.5)	70.3 (66.2, 74.5)	60.7 (56.2, 65.2	49.8 (45.1, 54.5)
No	68.3 (59.4, 77.2)	68.6 (59.5, 77.6)	61.0 (52.0, 69.9)	45.2 (36.2, 54.3)

Notes: Level of statistical significance: * p < 0.05; ** p < 0.01; *** p < 0.001. ^‡ ^Population level frequencies do not agree with Table 3 as don't know/refused responses were excluded from this analysis. ^§ ^For pairwise comparison testing in subgroups with more than two categories comparisons are made between each subgroup prevalence and the overall population prevalence. ^ψ^Psychological distress was measured using the K10. Values range from 10–50, with 'high' psychological distress considered as being ≥ 22.

Source: New South Wales Health Survey Program. Sydney: New South Wales Department of Health, 2008.

**Figure 2:**
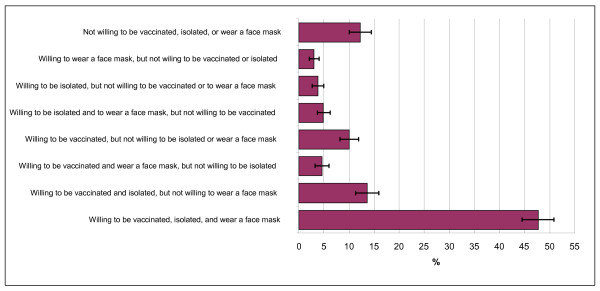
**Prevalence estimates and 95% confidence intervals for response combinations to the three questions on health protection behaviours for pandemic influenza**. Source: New South Wales Health Survey Program. Sydney: New South Wales Department of Health, 2008.

When the indicators for willing to receive vaccination, isolate themselves, and wear a face mask were combined, as shown in Figure 2, 48.3% reported being willing to receive vaccination, isolate themselves, and wear a face mask if pandemic influenza were to occur; 13.7% would not be willing to receive vaccination, isolate themselves and wear a face mask; 13.1% would be willing to receive vaccination, isolate themselves but not wear a face mask; and 10.4% would be willing to receive vaccination and wear a face mask but not isolate themselves.

Table 5 also shows the combined indicator for willing to receive vaccination, isolate themselves, and wear a face mask by sex, age group, demographic characteristics, and the indicators of level of psychological distress and general self-rated health status.

Table 6 shows the indicators for willing to receive vaccination, isolate themselves, or wear a face mask as well as complying with all the specific public health behaviours: that is, willing to receive vaccination, isolate themselves, and wear a face in people who think a pandemic influenza is very or extremely likely, and who are also very or extremely concerned for themselves and their family.

**Table 6:** Prevalence estimates and pairwise comparisons, willingness to comply with health protective behaviours in persons who think pandemic influenza is very or extremely likely to occur and are very or extremely concerned for self and family, by socio-demographic subgroups and health status

**Responses ONLY for those people who think that Pandemic influenza is very/extremely likely and are also very/extremely concerned for self and family (n = 212)**	**Very willing to comply with vaccination**	**Very willing to comply with isolation**	**Very willing to comply with wearing a face mask**	**Very willing to comply with ALL: vaccination + isolation+ wearing a face mask**
	**% (95%CI)**	**% (95%CI)**	**% (95%CI)**	**% (95%CI)**

**Total NSW population^‡^**	89.0 (84.2, 93.9)	80.2 (72.6, 87.8)	76.4 (69.1, 83.7)	63.6 (54.5, 72.8)

**Gender**				
Male	90.5 (83.0, 98.0)	80.6 (68.4, 92.8)	74.0 (61.6, 86.4)	64.8 (49.6, 80.0)
Female	87.6 (81.6, 93.7)	79.8 (70.3, 89.4)	78.6 (70.4, 86.8)	62.5 (52.3, 72.8)

**Age^§^**				
16–24	67.1 (23.0, 100)	**26.3 **(0, 71.1)*	52.6 (1.2, 100)	-
25–34	95.0 (85.0, 100)	59.9 (28.1, 91.7)	80.7 (60.0, 100)	51.9 (20.3, 83.6)
35–44	85.4 (68.9, 100)	86.0 (70.6, 100)	63.2 (35.5, 90.9)	55.8 (24.7, 87.0)
45–54	90.9 (84.0, 97.8)	76.2 (59.0, 93.4)	83.3 (72.6, 93.9)	65.0 (48.0, 82.0)
55–64	90.8 (83.0, 98.6)	**91.9 **(84.4, 99.5)*	77.9 (65.2, 90.6)	70.1 (55.6, 84.7)
65–74	92.4 (85.8, 99.0)	86.1 (76.8, 95.4)	85.9 (73.4, 98.4)	**81.8 **(71.5, 92.1)**
75+	87.1 (70.8, 100)	85.6 (68.1, 100)	77.2 (56.5, 97.9)	72.9 (50.9, 94.9)

**Children in household**				
Yes	93.6(84.8, 100)	71.7 (50.2, 93.3)	77.2 (60.7, 93.8)	58.9 (34.8, 83.0)
No	87.4 (80.9, 94.0)	77.0 (76.6, 90.8)	77.9 (68.9, 87.0)	69.6 (60.0, 79.3)

**Born in Australia**				
Yes	88.9 (83.3, 94.6)	84.9 (78.7, 91.1)	76.1 (67.8, 84.3)	68.0 (58.6, 77.5)
No	89.4 (80.1, 98.6)	64.2 (42.5, 85.9)	77.3 (62.0, 92.5)	48.6 (28.4, 68.8)

**Speak language other than English at home**				
Yes	84.6 (65.1, 100)	**41.1**(11.9, 70.2)	60.4 (32.8, 88.1)	**17.6 **(0, 35.9)
No	89.8 (85.4, 94.3)	**85.9 **(80.5, 91.4)**	79.2 (72.3, 86.1)	**70.4 **(62.2, 78.6)***

**Location**				
Urban	87.9 (80.3, 95.5)	81.3 (69.7, 92.8)	79.0 (68.8, 89.1)	63.5 (49.2, 77.8)
Rural	90.7 (85.9, 95.4)	78.8 (70.3, 87.4)	72.7 (63.2, 82.2)	63.8 (54.1, 73.5)

**Living alone**				
Yes	86.2 (77.7, 94.6)	84.3 (74.7, 93.9)	68.9 (55.5, 82.3)	51.6 (37.9, 65.3)
No	89.6 (84.1, 95.0)	79.5 (70.7, 88.3)	77.7 (69.5, 85.9)	65.8 (55.5, 76.2)

**Highest formal qualification^§^**				
None	92.1 (83.9, 100)	79.2 (64.9, 93.5)	65.0 (44.2, 85.8)	55.8 (34.1, 77.5)
School certificate	79.3 (66.7, 91.8)	73.9 (60.2, 87.7)	71.7 (57.9, 85.5)	55.1 (40.8, 69.5)
High school certificate	91.5 (82.0, 100)	75.8(50.0, 100)	86.3 (71.7, 100)	63.6 (38.8, 88.5)
TAFE certificate/diploma	87.0 (73.6, 100)	77.3 (61.6, 93.1)	68.0 (49.8, 86.2)	65.0 (46.5, 83.6)
University degree/equivalent	95.0 (89.9, 100)	88.6 (74.3, 100)	84.4 (73.4, 95.5)	71.7 (54.4, 90.0)

**Employed**				
Yes	88.5 (81.0, 96.0)	78.0 (67.0, 89.0)	74.9 (64.5, 85.3)	61.2 (48.9, 73.5)
No	88.3 (81.7, 95.0)	79.3 (67.9, 90.7)	74.1 (64.2, 84.0)	60.3 (48.3, 72.4)

**Household income^§^**				
<$20 k	94.1 (89.5, 98.7)	89.4 (81.8, 97.0)	75.3 (62.2, 88.4)	71.0 (57.6, 84.5)
$20 k – $40 k	79.8 (67.1, 92.4)	82.4 (69.7, 95.1)	81.2 (68.4, 94.0)	65.1 (50.1, 80.1)
$40 k – $60 k	86.7 (68.5, 100)	72.3 (53.1, 91.4)	64.4 (42.6, 86.3)	54.6 (33.4, 75.7)
$60 k – $80 k	93.4 (82.8, 100)	81.1 (51.8, 100)	86.0 (67.2, 100)	68.3 (32.6, 100)
> $80 k	93.5 (85.9, 100)	91.5 (81.3, 100)	83.3 (69.1, 97.5)	73.1 (55.6, 90.5)

**High psychological distress (≥ 22)^ψ^**				
Yes	90.1 (75.3, 100)	75.2 (47.0, 100)	85.8 (71.2, 100)	57.2 (29.3, 85.1)
No	85.6 (4.4, 77.0)	81.0 (71.7, 90.2)	69.4 (57.8, 81.1)	61.7 (49.7, 73.6)

**Positive self-rated health status (good, very good, excellent)**				
Yes	93.6 (89.0, 98.2)	86.4 (77.8, 95.1)	79.4 (68.5, 90.4)	**74.0 **(61.6, 86.4)*
No	90.1 (80.2, 99.9)	64.0 (39.6, 88.4)	67.7 (46.3, 89.0)	**40.3 **(16.8, 63.8)

Notes: Level of statistical significance: * p < 0.05; ** p < 0.01; *** p < 0.001. ^‡ ^Population level frequencies do not agree with Table 3 as don't know/refused responses were excluded from this analysis. ^§ ^For pairwise comparison testing in subgroups with more than two categories comparisons are made between each subgroup prevalence and the overall population prevalence. ^ψ^Psychological distress was measured using the K10. Values range from 10–50, with 'high' psychological distress considered as being ≥ 22.

Source: New South Wales Health Survey Program. Sydney: New South Wales Department of Health, 2008.

## Discussion

This study shows it is possible to construct a small set of questions about threat perception for a general population, which can be used for health surveillance. Field testing identifies improvements that can be made to the questions and the response structure, and highlights the population's interest in surveys of this nature. Kappa values for the indicators ranged from 0.25–0.51, which is acceptable for hypothetical questions. The items had low don't know response rates (0–3.9%); no respondents refused to answer any of the questions; and the majority of respondents made positive comments about the questions.

Those reporting the highest levels of threat perception are older people, those with fair or poor self-rated health status, no formal qualifications, low household incomes, and those living in rural areas. Perhaps surprisingly, there were no differences noted between the perceptions of men and women, or between those persons with or without children.

Overall, the majority of the population has taken no action, at this point, to change the way they live their life because of the possibility of pandemic influenza. The only two subgroups reporting moderate changes are those born overseas and those who speak a language other than English in the home.

Although direct comparisons with other studies are difficult to make, these findings suggest that the threat perceptions of the New South Wales population are similar to those reported by residents of Hong Kong, even though Australia has not been exposed directly to SARS or H5N1.

Willingness to comply with specific public health behaviours is generally high (60–75%), with willingness to be vaccinated greater than being willing to be isolated, which in turn is greater than being willing to wear a face mask. There is clearly a lower level of willingness to comply with wearing a face mask, especially in younger people, those living in urban areas, and those who speak a language other than English in the home.

Current findings on compliance with protective behaviours are comparable with findings from studies conducted in Hong Kong in relation to anticipated SARS and H5N1.[4,7] A study about SARS in Hong Kong indicates that those with higher risk perception and moderate levels of anxiety were more likely to take comprehensive precautionary measures against infection, and younger less educated males were least likely to adopt preventative measures.[3] Our data suggest that younger people are less likely to comply with protective behaviours, while a higher level of formal education (a university degree or equivalent) is associated with higher willingness to comply with all protective behaviours, but especially wearing a face mask.

A study of this nature has a number of limitations. First, people are being asked about a hypothetical event of which they have no experience. However, comparisons with other studies, where respondents have direct experience of real events, suggest similar patterns of response. Second, the actual level of compliance with protective behaviours correlates with an actual and immediate threat. For example, Lau et al. plotted changes in mask wearing behaviour during an outbreak of SARS in Hong Kong in 2003,[4] and reported mask wearing rising from 11% in the early stages to 94% in the later stages of the outbreak. Clearly data in that study support the increased likelihood of protective behaviours being adopted with increased risk perception; and, in our study, those with higher levels of threat perception were significantly more likely to be willing to comply with specific public health behaviours.

Our data indicate that while most respondents are very or extremely willing to perform a behaviour; the remaining respondents are expressing varying, but lower, degrees of willingness to perform these behaviours, with 21–31% indicating they would be moderately or a little willing, and 3–8% indicating they would be not at all willing to perform these behaviours. However, evidence such as data indicating very high levels of compliance with quarantine and minimal requirement for enforceable quarantine orders during SARS in Canada suggests that, in the event of a serious and immediate threat, the majority of those who are indecisive would shift their position and comply.[19] It is likely, however, that even with such a compliance 'shift' the relative compliance of sub groups within the population noted in our study will be upheld; as these patterns of compliance have been supported consistently by studies of actual protective behaviours.[3,4]

## Conclusion

This study of the response of the New South Wales population to the threat of pandemic influenza is part of a broader study of perceptions and behaviours around adverse events, including terrorism and global warming. As post-disaster studies generally report a lack of baseline data as a major handicap to understanding the trajectory for psychosocial recovery,[17,18] our study takes the first steps in establishing baseline for data vital for emergency planning, against which impact and recovery can be monitored.

## Competing interests

The authors declare that they have no competing interests.

## Authors' contributions

The authors contributed equally to this work.

## Pre-publication history

The pre-publication history for this paper can be accessed here:



## References

[B1] Commonwealth of Australia (2006). Australian Health Management Plan for Pandemic Influenza.

[B2] Smith RD (2005). Infectious disease and risk: Lessons from SARS.

[B3] Leung GM, Lam TH, Ho LM, Ho SY, Chan BH, Wong IO, Hedley AJ (2003). The impact of community psychological responses on outbreak control for Severe Acute Respiratory Syndrome in Hong Kong. J Epidemiol Community Health.

[B4] Lau JTF, Yang X, Tsui H, Kim JH (2003). Monitoring community psychological responses to the SARS epidemic in Hong Kong: From day 10 to day 62. J Epidemiol Community Health.

[B5] Cava MA, Fay KE, Beanlands HJ, McCay EA, Wignall R (2005). Risk perception and compliance with quarantine during the SARS outbreak. J Nurs Scholarsh.

[B6] Fielding R, Lam WW, Ho EY, Hedley AJ, Leung GM (2005). Avian influenza risk perception: Hong Kong. Emerg Infect Dis.

[B7] Lau JTF, Kim JH, Tsui HY, Griffiths S (2007). Anticipated and current preventative behaviours in response to an anticipated human-to-human H5N1 epidemic in Hong Kong Chinese general population. BMC Infectious Diseases.

[B8] De Zwart O, Veldhuijzen IK, Elam G, Aro AR, Abraham T, Bishop GD, Richardus JH, Brug J (2007). Avian Influenza Risk Perception, Europe and Asia. Emerg Infect Dis.

[B9] Di Giuseppe G, Abbate R, Albano L, Marinelli P, Angelillo IF (2008). A survey of knowledge, attitudes and practices towards avian influenza in an adult population of Italy. BMC Infect Dis.

[B10] Lemyre L, Lee JEC, Krewski D (2004). National public survey of perceived CBRN terrorism threat and preparedness. University of Ottawa in partnership with Health Canada and the Canadian Food Inspection Agency.

[B11] Barr M, Baker D, Gorringe M, Fritsche L (2008). NSW Population Health Survey: Description of Methods.

[B12] Australian Bureau of Statistics (1996). Population Survey Monitor.

[B13] Brick JM, Dipko S, Presser S, Tucker C, Yuan Y (2005). Estimation Issues in Dual Frame Sample of Cell and Landline Numbers; ASA Section on Survey Research Methods.

[B14] Yuan Y, Allen B, Brick JM, Dipko S, Presser S, Tucker C, Han D, Burns L, Galesic M (2005). Surveying households on cell phones: Results and lessons. Paper presented at the Annual Conference of the American Association for Public Opinion Research, Miami, Florida.

[B15] Steel D (2008). NSW Population Health Survey: Review of Weighting Procedures.

[B16] Australian Bureau of Statistics (2006). Census of Population and Housing.

[B17] Lerner JS, Gonzalez RM, Small DA, Fischhoff B (2003). Effects of fear and anger on perceived risks of terrorism: A national field experiment. Psychol Sci.

[B18] North CS, Pfefferbaum B, Narayanan P, Thielman S, McCoy G, Dumont C, Kawasaki A, Ryosho N, Spitznagel EL (2005). Comparison of post-disaster psychiatric disorders after terrorist bombings in Nairobi and Oklahoma City. Br J Psychiatry.

[B19] Svoboda T, Henry B, Shulman L, Kennedy E, Rea E, Ng W, Wallington T, Yaffe B, Gournis E, Vicencio E, Basrur S, Glazier R (2004). Public health measures to control the spread of the Severe Acute Respiratory Syndrome during the outbreak in Toronto. N Engl J Med.

